# The Association between Phthalates in Dust and Allergic Diseases among Bulgarian Children

**DOI:** 10.1289/ehp.10498

**Published:** 2007-10-15

**Authors:** Barbara Kolarik, Kiril Naydenov, Malin Larsson, Carl-Gustaf Bornehag, Jan Sundell

**Affiliations:** 1 Technical University of Denmark, Department of Mechanical Engineering, International Centre for Indoor Environment and Energy, Lyngby, Denmark; 2 Silesian University of Technology, Faculty of Environmental Engineering and Energy, Gliwice, Poland; 3 Birch & Krogboe A/S, Virum, Denmark; 4 Public Health Sciences, Karlstad University, Karlstad, Sweden; 5 SP-Technical Research Institute of Sweden, Boras, Sweden

**Keywords:** allergy, asthma, children, DEHP, phthalates

## Abstract

**Background:**

Recent studies have identified associations between the concentration of phthalates in indoor dust and allergic symptoms in the airways, nose, and skin.

**Objectives:**

Our goal was to investigate the associations between allergic symptoms in children and the concentration of phthalate esters in settled dust collected from children’s homes in Sofia and Burgas, Bulgaria.

**Methods:**

Dust samples from the child’s bedroom were collected. A total of 102 children (2–7 years of age) had symptoms of wheezing, rhinitis, and/or eczema in preceding 12 months (cases), and 82 were nonsymptomatic (controls). The dust samples were analyzed for their content of dimethyl phthalate (DMP), diethyl phthalate (DEP), di-*n*-butyl phthalate (DnBP), butyl benzyl phthalate (BBzP), di(2-ethylhexyl) phthalate (DEHP), and di-*n*-octyl phthalate (DnOP).

**Results:**

A higher concentration of DEHP was found in homes of case children than in those of controls (1.24 vs. 0.86 mg/g dust). The concentration of DEHP was significantly associated with wheezing in the preceding 12 months (*p* = 0.035) as reported by parents. We found a dose–response relationship between DEHP concentration and case status and between DEHP concentration and wheezing in the preceding 12 months.

**Conclusions:**

This study shows an association between concentration of DEHP in indoor dust and wheezing among preschool children in Bulgaria.

In the developed parts of the world, people spend ≥ 90% of their life indoors (Brasche and Bischof 2005), which implies that indoor environmental conditions are important for people’s health. Indoors, pollutants in dust and air are often generated from sources such as environmental tobacco smoke, building materials, furniture, cleaning and hygienic products, air fresheners, computers, printers, cooking and other indoor activities, and from people themselves.

Over the last few decades, asthma and allergies have increased all over the world [Asher et al. 2006; ISAAC (International Study of Asthma and Allergy in Childhood) Steering Committee 1998; World Health Organization 2003]. The causes of the increase in asthma and allergies are still unknown. Genetic changes are not believed to be important because the time interval (30–50 years) for the increase in allergies is far too short. Instead, environmental changes are suspected as possible causes. Several hypotheses have been put forward. Much interest has been focused on the so called “hygiene hypothesis”—that a lack of microbial exposure during critical periods in infancy increases the risk of allergies (Strachan 1989). However, it seems likely that this hypothesis may not be the sole cause, and that other hypotheses are required (Platts-Mills et al. 2005). One such hypothesis is that the increase in allergies is attributable to new adjuvant factors: exposure to environmental pollutants such as endocrine disruptors (e.g., phthalate esters) which may act as modulators of the immune system and induce an allergic response (Chalubinski and Kowalski 2006).

One of the main sources for phthalate esters indoors is the plasticized polyvinyl chloride (PVC) materials (Bornehag et al. 2005a) that are used in floor and wall covering materials, shower curtains, adhesives, synthetic leather, toys, cosmetics, and many other consumer products. Phthalates are constantly being emitted to the air and indoor dust because they are not chemically bound to the PVC structure (Wormuth et al. 2006).

The presence of phthalates in indoor dust (Becker et al. 2004; Bornehag et al. 2004; Butte et al. 2001; Clausen et al. 2003; Fromme et al. 2004; Kersten and Reich 2003; Oie et al. 1997; Pohner et al. 1997; Rudel et al. 2003) and in indoor air (Adibi et al. 2003; Fromme et al. 2004; Rudel et al. 2003) is well documented. In the literature, the predominant phthalate described in indoor dust is di(2-ethylhexyl) phthalate (DEHP), typically observed in a concentration range of 0.01–10 mg/g dust, followed by butyl benzyl phthalate (BBzP) in concentrations up to 1.3 mg/g dust. The phthalate concentration in indoor air is usually lower than the concentration in dust, and the predominant phthalates are diethyl phthalate (DEP) and di-*n*-butyl phthalate (DnBP) in the concentration range of 0.05–5 μg/m^3^. Human exposure to phthalates has been studied mainly by monitoring concentrations of metabolites in body fluids such as urine or blood (Adibi et al. 2003; Becker et al. 2004; Blount et al. 2000; Calafat et al. 2004; Green et al. 2005; Koch et al. 2003, 2005). The results have shown that people are exposed to multiple phthalates and that children often are more exposed than adults (Calafat et al. 2004; Green et al. 2005; Koch et al. 2005).

There is some epidemiologic evidence for an association between the concentration of phthalates in indoor dust and/or the occurrence indoors of plasticized products such as PVC and allergic symptoms in the airways (e.g., asthma), nose, and skin. Jaakkola et al. (1999) found that the total area of PVC surface materials in homes was associated with the development of bronchial obstruction in small children in Norway. In a study from Finland (Jaakkola et al. 2000), lower respiratory tract symptoms in children such as persistent wheezing, cough, and phlegm were associated with the presence of plastic wall materials, whereas upper respiratory tract symptoms were not. Also, the relative risk estimated for pneumonia, bronchitis, and otitis media among children was slightly increased in the presence of plastic wall materials (Jaakkola et al. 2000). In a population-based incident case–control study among adults (21–63 years of age), Jaakkola et al. (2006) found that the risk of asthma was significantly related to the presence of plastic wall materials at work.

In the first phase of the Swedish DBH (Dampness in Buildings and Health) study it was found that PVC as flooring material in combination with moisture problems in the floors was associated with asthma among children 1–6 years of age (Bornehag et al. 2005b). Furthermore, in the second phase of the DBH study a strong dose–response relationship was found between asthma among children and DEHP concentration in indoor dust and between eczema and rhinitis and BBzP (Bornehag et al. 2004). Oie et al. (1997) provided evidence that inhalation exposure to DEHP as aerosols adsorbed to particulate matter is even more important than vapor phase exposure. They discussed possible mechanisms for respiratory effects by inhalation exposure and concluded that deposition of DEHP in the lungs may increase the risk of inflammation in the airways, a characteristic feature of asthma.

The results from toxicologic studies are conflicting. Lee et al. (2004) reported that DEHP and di-isononyl phthalate (DINP) enhance allergic responses by enhancement of interleukin (IL)-4 production in CD4^+^ T cells via stimulation of NF-AT (nuclear factor of activated T cells)–binding activity. Glue et al. (2005) investigated the effect of phthalates to modulate the release of histamine from isolated basophils. None of the phthalates tested was found to induce histamine release per se, but higher histamine release was observed when the cells were first treated with phthalates and then exposed to an allergen. Recently, Larsen et al. (2007) reported that long-term inhalation of DEHP together with an allergen resulted in allergy sensitization only in concentrations of 13 mg/m^3^. These authors concluded that DEHP, at realistic concentrations, does not cause adjuvant effects nor allergic lung inflammation in humans. In a recent review, Nielsen et al. (2007) concluded that results from animal and epidemiologic studies are discordant.

This study (The ALLHOME study) was initiated in Bulgaria in 2004. The overall aim of the study was to map housing conditions and indoor exposures in Bulgaria and to investigate the role of such factors for allergies and asthma among small children (Naydenov 2007; Naydenov et al. 2005).

Our main aim in the present article is to investigate associations between persistent allergic symptoms in preschool Bulgarian children and the concentrations of different phthalate esters in dust collected from the children’s bedrooms.

## Methods

The ALLHOME study is divided into two phases: a cross-sectional questionnaire study (ALLHOME-1) and a nested case–control study (ALLHOME-2), including dwelling inspections, exposure measurements, and medical examinations. The ALLHOME study was designed to be a twin study to the Swedish DBH study (Bornehag et al. 2004; Naydenov 2007).

From April 2004 to August 2004 a baseline questionnaire (ALLHOME-1) on housing and health was sent to the parents of all children 2, 3, 5, and 7 years of age living in selected districts of Sofia and Burgas, Bulgarian cities with populations of about 1.2 million and 0.2 million, respectively. Data for 4,479 children was collected, corresponding to a response rate of 34.5%. Based on reported symptoms in the ALLHOME-1 study, potential case and control children in the ALL-HOME-2 study were selected (Naydenov 2007). Potential cases were all children who were reported to have at least two of the following three symptoms in the ALLHOME-1 study: “wheezing during the last 12 months,” “rhinitis during the last 12 months, when not having a cold,” and “itching rash eczema in the last 12 months.” The potential controls were all children who reported an absence of all three symptoms stated above as well as absence of “wheezing ever in the past,” “dry cough more than 2 weeks, without a cold,” “diagnosed asthma by a doctor ever in the past,” “running nose ever in the past without cold/flu,” “running nose or watering eyes on pet contact in the last 12 months,” “running nose or watering eyes on pollen contact in the last 12 months,” “diagnosed hay fever by a doctor ever in the past,” and “itching rash ever in the past lasting more than 6 months in typical locations of the child’s body.” No matching of cases or controls was carried out.

Of 4,479 children, 2,105 met the inclusion criteria for cases and controls: 730 (16.3%) were potential cases and 1,375 (30.7%) were potential controls. In a follow-up questionnaire study (3 months later), parents of both cases and controls had to agree to participate, had to have not rebuilt their house because of moisture problems, and had to have not changed home during the time between the baseline and follow-up questionnaire. Furthermore, invited case children had to have at least one of the three symptoms (wheezing, rhinitis, eczema), and invited controls had to be free from the symptoms listed in the follow-up questionnaire. The selection procedure identified a total of 272 children (136 cases and 136 controls) (Naydenov 2007).

Exposure measurements in the homes and health examinations of the case and control children were conducted from December 2004 to March 2005. Despite their written consent given in the follow-up study, the parents of 56 children refused to have their home inspected. Home investigations were therefore performed in 209 homes of 216 children because there were seven pairs of siblings. In 26 of the inspected homes, inspectors were not allowed to perform dust sampling; thus dust samples from only 183 homes of 190 children were collected. Six samples were either inadvertently destroyed in the laboratory or not coded by inspectors. As a result, dust samples from 177 homes of 184 children, including 102 cases and 82 controls, were available for analysis.

Sampling of settled dust was performed in the child’s room, above the floor level, such as over the door and from shelves and frames of paintings. A 1,600-W vacuum cleaner equipped with a phthalate-free ALK dust sampling device (ALK dust collector and filter; ALK-Abelló A/S, Hørsholm, Denmark) was used for the dust collection. The dust samples were wrapped in aluminum foil and kept in a polyethylene bag with a zip lock, so that the dust had no contact with the bag. Samples were frozen the day of sampling at −18°C. They were later thawed to room temperature and sent to a laboratory. The dust was not sieved, but the filter used retained 74% of particles 0.3–0.5 μm, 81% of particles 0.5–1.0 μm, 95% of particles 1–10 μm, and 100% of larger particles (ISAAC 1998). The filters were not weighed before the sampling took place, but the dust was weighed before analysis at room temperature. The phthalate concentrations are expressed as a fraction of the dust that could be shaken out of the filter. Dust samples were extracted in 2-mL glass vials for 1 hr using 1 mL of 10% toluene solution in carbon disulfide. Two milliliters of this solution was injected into a capillary column (Hewlett Packard DB-5 capillary column, H-30 m, 0.53 mm diameter). The column temperature started at 80°C for 5 min and then increased at 20°C/min to 290°C, which was maintained for 10 min. The technique used for the analysis was gas chromatography/ flame ionization detection, which is used for qualitative identification and quantitative determination. In this method, components separated in the gas chromatograph pass through the flame, burn, and produce ions, with a current that is proportional to the amount of hydrocarbons in the sample. Six phthalates were analyzed: dimethyl phthalate (DMP), DEP, DnBP, BBzP, DEHP, and di*n*-octyl phthalate (DnOP). The detection limits, in milligrams of phthalate per gram of dust (for median dust weight), was in the range of 0.06–0.26 mg/g, depending on the type of phthalate. DEHP and DnBP were found in all analyzed samples. For other phthalates, the percent of nondetects is given in Table 2.

### Statistical analysis

The analyses of concentrations of phthalates in dust and building characteristics were made for all 177 homes visited. The concentrations of phthalates were not normally distributed. The concentrations are reported as medians, arithmetic means, and geometric means (GM) with 95% confidence intervals (CI) where the CI was calculated with a back-transform of mean log ± 2 × SE. Ranges of concentrations of measured phthalates are also presented.

We analyzed potential associations between concentration of phthalates and health outcomes using the nonparametric Mann-Whitney *U*-test and one-way analysis of variance and Dunnett’s test (on log-transformed data). Dunnett’s test controls for family-wise error rate (which is the probability of making false discoveries, or type I errors among all the hypotheses when performing multiple pairwise tests) and can be used for multiple comparisons, when comparing with controls.

We tested dose–response relationships using the phthalate concentrations in quartiles and using both uni- and multivariate logistic regression analyses. We tested dose–response relationships with a trend test. Results are presented as crude and adjusted odds ratios (ORs) with 95% CIs. In all multivariate analyses, adjustments were made for age (< 3 years vs. ≥ 5 years of age), sex (male vs. female), smoking during pregnancy and first year of child’s life (any parents smoking in the house during the pregnancy and first year of the child’s life vs. no smoking in the home), current smoking at home (one or more occupants currently smoking in the house vs. no smoking) and allergy and/or asthma symptoms in the family (at least one symptomatic family member other than the index child vs. no other person with symptoms). Because all the included siblings (*n* = 7) had separate rooms and the separate dust samples had been collected for each of them, the analyses of association between phthalates and health were made for 184 children. Additional analyses, limited to one child per household (selected based on lower identification number) were also performed.

All analyses were made by using SPSS 15.0 for Windows (SPSS Inc., Chicago, IL, USA).

The study was approved by the local ethics review board. Parents gave written informed consent to the participation of their children in the study.

## Results

The study group consisted of 96 boys and 88 girls, 105 of them < 3 years of age. Nine families lived in single-family houses, the rest in multifamily houses, and 131 (75%) of the houses were built in 1960–1990. All buildings had natural ventilation, and 40% had a kitchen exhaust hood with a fan. Table 1 shows selected demographic, building and family routine characteristics.

### Phthalates in dust

Geometric mean phthalate concentrations with 95% CIs, in milligrams of phthalate per gram of dust, are shown in Table 2. For most of the samples analyzed, phthalates were above the detection limits. DEHP and DnBP were found in all collected samples.

### Associations between concentrations of phthalates in dust and health status of the children

Median and arithmetic mean phthalate concentrations for case and control children are presented in Table 3. In general, there were no differences in the concentration of most phthalates in dust between homes of cases and controls. However, DEHP was found in higher concentrations in the homes of cases than in controls. Furthermore, case children with reported wheezing in the preceding 12 months had significantly higher concentrations of DEHP than controls. The concentration of BBzP was higher in the homes of children with wheezing and eczema (both in the preceding 12 months), though not significantly higher.

A significant dose–response relationship was observed between the concentration of DEHP in indoor dust and case status, and between DEHP and current wheezing (Table 4).

In Bulgaria, PVC and linoleum floor materials are sold under the common name *balatum*, which makes it impossible to distinguish reliably between them based on information obtained from parental questionnaires or inspectors’ notes. However, the presence of *balatum* in the child’s bedroom, as reported by inspectors, was significantly associated with case status among the children (adjusted OR = 2.21; 95% CI, 1.13–4.32). A significant association was also found between *balatum* (as reported by inspectors) and both wheezing (adjusted OR = 2.64; 95% CI, 1.34–5.21) and rhinitis in preceding 12 months (adjusted OR = 2.15; 95% CI, 1.08–4.17). The same results were obtained for the presence of *balatum* as reported by parents in the main questionnaire and health effects. There was no association between concentration of phthalates in dust and the presence of *balatum* flooring.

Polishing products, used when dusting the furniture, were found to be a strong source of phthalates in Bulgaria. The use of polishing products in the home (at least once per month compared with no use or very rare use), as reported by parents, was associated with case status (adjusted OR = 1.75; 95% CI, 0.95–3.23), wheezing in the preceding 12 months (adjusted OR = 1.92; 95% CI, 1.02–3.62), and rhinitis in the preceding 12 months (adjusted OR = 1.71; 95% CI, 0.90–3.23). The use of polishing products was also significantly associated with the concentration of BBzP (*p* = 0.025) and DnOP (*p* = 0.040) in indoor dust. Concentrations of DEP and DEHP were also higher, though not significantly higher, in homes where such products were used. A high frequency of cleaning (dusting) of furniture was associated with fewer health problems (as defined by case status and by all three symptoms) but with concentration of BBzP, which was significantly higher in homes that were cleaned more often (*p* = 0.007).

## Discussion

The concentration of DEHP in indoor dust was in about the same concentration range as in several other studies, including the Swedish DBH study (Bornehag et al. 2004) (Table 5). The concentration of BBzP was found in somewhat higher concentration in Bulgaria compared with other studies. Large differences were observed for DnBP, which was found in concentrations up to 40 times higher in Bulgaria than in other countries (Table 5), and for DEP and DMP, which were also found in much higher concentrations in the present study than in other studies (Bornehag et al. 2004; Fromme et al. 2004). None of the main sources identified (*balatum* flooring, polishing products) were correlated with the concentration of DnBP, and only a slight, nonsignificant correlation was found between DEP and use of polishing products. No information on other possible sources of these phthalates (e.g. cosmetics, soft toys, plastic covers on furniture) was obtained, so we are unable to put forward any hypotheses that might explain the high concentrations of DnBP and DEP that were found in this study.

The results support the finding from the Swedish DBH study regarding an association between DEHP and asthmatic and allergic symptoms among children (Bornehag et al. 2004). Although the Swedish findings regarding an association of the dust concentration of BBzP with rhinitis and eczema were not replicated in a significant manner in the current study, the results from Bulgaria point to the same conclusion as in Sweden.

In the present study, each child was treated as separate observation, because in homes with multiple children participating in the study, each child had a separate bedroom, so a separate dust sample was collected. However, all analyses performed on only one child per home (177 children) gave corresponding results, with even stronger significance (data not shown).

In contrast to the results of the Swedish DBH study (Bornehag et al. 2005a), we found no correlation between concentrations of DEHP and BBzP in dust and the presence of *balatum* in children’s sleeping rooms. One explanation can be that in Bulgaria the word *balatum* has two meanings: linoleum or PVC. The fact that some of the “plastic” flooring was in fact linoleum can therefore have weakened or even removed any possible correlation between PVC flooring and phthalate concentration. For both studies, however, an association between health and the presence of PVC/*balatum* flooring in the child’s room was observed. In the present study, of 60 homes with *balatum* flooring (34% of investigated homes), 41 were found in the homes of case children (Table 1).

Because the use of polishing agents and the concentration of phthalates are correlated with each other and both of them were associated with symptoms, the association between health outcomes and DEHP concentration was tested in both adjusted and stratified analyses. We found a dose–response relationship between DEHP in dust and symptoms both in buildings with high and low polish use, with ORs in the highest quartiles (compared with the lowest quartile group): OR = 4.20 (95% CI, 0.96–18.33) for case status, and OR = 3.73 (95% CI, 0.85–16.44) for wheezing in the group of homes with high polish use; and OR = 1.93 (95% CI, 0.62–5.98) for case status and OR = 2.67 (95% CI, 0.79–8.95) for wheezing in the low polish use group. Also, with adjusting for polish use, the significant association between the concentration of DEHP and case status/ wheezing were found for the highest quartile (compared with the lowest quartile group): OR = 2.61 (95% CI, 1.07–6.37) for case status, and OR = 3.08 (95% CI, 1.21–7.83) for wheezing in preceding 12 months. This means that the association between phthalates and health cannot be explained by different use of polishing agents only; however, the use of such compounds seems to reinforce the association between health and the concentration of selected phthalates.

It is reasonable to expect that case families with asthma and allergy clean more frequently than control families, which could increase the amount and age of the indoor dust and thus the content of phthalates in control homes. In this study we found no difference in cleaning frequency in homes of cases and controls, and we found the opposite in dusting furniture—namely, that controls clean more frequently than case families. Parents of 44% of controls and 32% of cases reported that they customarily clean their furniture more often than once a week. However, adjusting for dusting frequency did not change the results in this study. Additionally, the only phthalate significantly associated with low dusting frequency was BBzP, which was found in significantly higher concentrations in homes where dusting was carried out once a week or less often, compared with more frequent dusting (*p* = 0.007). DEHP and DnOP were also found in higher concentrations in homes where dusting was not as frequent, compared with homes with more frequent dusting, although this difference was not significant.

Compared with Sweden, there were numerous problems in conducting the study in Bulgaria. The response rate in the cross-sectional study was very low (34.5%) compared with Sweden (almost 80%). In the current case–control study, several families refused to allow inspections or dust sampling, despite having given prior consent. These differences are most probably attributed to political and socioeconomic factors that are outside the scope of this study.

A low response rate is always a problem in epidemiologic investigations. However, such problems mainly involve representativity and may not introduce bias when analyzing associations between exposures and health effects. The baseline study was used mainly to select possible cases and controls (sick and healthy children).

Because it is known that families with allergic diseases are more prone to participate in epidemiologic studies, potential selection bias in the baseline questionnaire was investigated. A total of 240 children (78 in Burgas and 162 in Sofia) were randomly selected among nonresponders; they were contacted by phone and asked to answer all questions used in the questionnaire. Excluding eczema in preceding 12 months and asthma/allergic symptoms among family members, there was no difference between the children whose parents took part in the ALLHOME-1 study and those whose parents refused to do so, limiting the risk for serious selection bias regarding allergic and asthmatic symptoms and diagnoses (Naydenov 2007).

Among children whose parents agreed to participate in the ALLHOME-2 study, the prevalence of current wheezing, rhinitis, and eczema was higher compared with children whose parents did not agree to participate in the ALLHOME-2 study or did not reply in the follow-up study (Naydenov 2007). More health problems in the case families was one of the selection factors for participation in the Swedish DBH study (Bornehag et al. 2006). However, such selection bias results in a greater contrast in health status between cases and controls, and hence a greater possibility of identifying differences in health-relevant exposures (Bornehag et al. 2006).

In this study, we analyzed associations between children’s health (as reported by parents), and measured or inspected indoor environmental factors. A bias in the baseline study could be introduced if parents with sick children knew the risk factors for their child’s illness and reported more of such factors. However, such risk factors were measured or observed by “blinded” inspectors in the second phase. Also, the main potential risk factor studied—the concentration of specific phthalates—is not known to be a risk factor by the general public in Bulgaria, Sweden, or elsewhere. A strong source of such compounds, PVC flooring, could be recognized and reported, and thus be a proxy for phthalates. This was not possible in Bulgaria, where there is not even a word for PVC flooring, because *balatum* is used for both PVC and linoleum flooring. The same discussion is valid with regard to use of polish.

This study and the DBH study in Sweden both show an association between DEHP in indoor dust and airway symptoms in preschool children. The two studies were performed in very different regions of Europe with regard to building type, climate, and political and socioeconomic factors. The mechanisms behind these results are not known. Some toxicologic studies have indicated that DEHP may act as an adjuvant factor (Chalubinski and Kowalski 2006; Nielsen et al. 2007). However, it is impossible to determine whether the important exposure is during childhood or during pregnancy.

## Conclusions

The main finding of this study is that phthalates in indoor dust could be found in all samples from Bulgarian homes. The second main finding is that there is a significant association between the concentration of DEHP in indoor dust and wheezing among preschool children.

## Figures and Tables

**Table 1:** Data on buildings characteristic and habits of 177 visited families and comparison with whole study population (*n* = 4,479) children [no. (%)].

	177 visited homes			Whole population (total)
Characteristic	Total	Controls	Cases	
Sex				
Male	92 (52)	37 (47)	55 (56)	2,337 (52)
Female	85 (48)	42 (53)	43 (44)	2,142 (48)
Age				
< 3 years	100 (56)	50 (63)	50 (51)	2,369 (53)
> 3 years	77 (44)	29 (37)	48 (49)	2,110 (47)
Town				
Sofia	71 (40)	33 (42)	38 (39)	2,049 (46)
Burgas	106 (60)	46 (58)	60 (61)	2,396 (54)
Type of flooring				
*Balatum*	60 (34)^a^	19 (24)^a^	41 (42)^a^	1,506 (34)^a^
Other	106 (60)^a^	54 (68)^a^	52 (53)^a^	2,621 (59)^a^
Use of polishing agents				
Yes	76 (43)^a^	28 (35)^a^	48 (49)^a^	NA^b^
No	97 (55)^a^	49 (62)^a^	48 (49)^a^	NA^b^
Smoking in the past^c^				
Yes	128 (72)^a^	50 (63)	78 (80)^a^	3,174 (71)^a^
No	47 (27)^a^	29 (37)	18 (18)^a^	1,162 (26)^a^
Current smoking				
Yes	129 (73)	49 (62)	80 (82)	3,240 (72)
No	48 (27)	30 (38)	18 (18)	1,237 (28)
Allergy in family				
Yes	53 (30)^a^	14 (18)	39 (40)^a^	3,068 (68)^a^
No	123 (69)^a^	65 (82)	58 (59)^a^	1,400 (31)^a^

a The sum is < 100% because some families did not answer this question.

b Not applicable: no question about polishing agents was asked in the baseline questionnaire.

c Smoking during pregnancy or first year of child’s life.

**Table 2:** The concentrations of phthalates (mg phthalate per g dust) measured in 177 homes of Bulgarian children shown as geometric mean with 95% CIs.

Phthalate	No. of samples above detection limit	Nondetects (%)	Geometric mean (95% CI; range)
DMP	162	8.5	0.26 (0.21–0.32; 4.30)
DEP	174	1.7	0.35 (0.29–0.42; 9.07)
DnBP	177	0.0	7.86 (6.59–9.36; 58.07)
DEHP	177	0.0	0.96 (0.79–1.17; 29.44)
BBzP	170	4.0	0.32 (0.28–0.38; 2.73)
DnOP	143	19.2	0.25 (0.20–0.30; 2.51)

**Table 3:** Concentration of phthalates in surface dust for wheezing, rhinitis, and eczema symptoms, divided by case and control children.

	Median (mean) concentration of phthalates (mg/g dust)					
Phthalate	No.	Case	No.	Controls	*p-*Value^a^	*p-*Value^b^
DMP						
Case status	91	0.24 (0.48)	77	0.30 (0.60)	0.297	0.985
Wheezing preceding 12 months	77	0.23 (0.48)	77	0.30 (0.60)	0.200	0.994
Rhinitis preceding 12 months	79	0.23 (0.40)	77	0.30 (0.60)	0.195	0.995
Eczema preceding 12 months	38	0.29 (0.66)	77	0.30 (0.60)	0.762	0.608
DEP						
Case status	100	0.32 (0.68)	81	0.36 (0.74)	0.376	0.954
Wheezing preceding 12 months	86	0.31 (0.68)	81	0.36 (0.74)	0.334	0.963
Rhinitis preceding 12 months	84	0.30 (0.66)	81	0.36 (0.74)	0.356	0.955
Eczema preceding 12 months	43	0.35 (0.70)	81	0.36 (0.74)	0.665	0.891
DnBP						
Case status	102	9.61 (12.15)	82	9.87 (12.04)	0.749	0.584
Wheezing preceding 12 months	88	11.17 (12.79)	82	9.87 (12.04)	0.469	0.405
Rhinitis preceding 12 months	86	8.63 (10.69)	82	9.87 (12.04)	0.715	0.854
Eczema preceding 12 months	44	9.61 (13.30)	82	9.87 (12.04)	0.667	0.499
DEHP						
Case status	102	1.24 (2.29)	82	0.86 (1.81)	0.030	0.084
Wheezing preceding 12 months	88	1.75 (2.48)	82	0.86 (1.81)	0.009	0.035
Rhinitis preceding 12 months	86	1.03 (1.64)	82	0.86 (1.81)	0.190	0.415
Eczema preceding 12 months	44	0.93 (2.76)	82	0.86 (1.81)	0.273	0.216
BBzP						
Case status	100	0.38 (0.53)	77	0.32 (0.45)	0.349	0.374
Wheezing preceding 12 months	87	0.38 (0.53)	77	0.32 (0.45)	0.305	0.336
Rhinitis preceding 12 months	84	0.32 (0.49)	77	0.32 (0.45)	0.630	0.583
Eczema preceding 12 months	43	0.40 (0.60)	77	0.32 (0.45)	0.207	0.213
DnOP						
Case status	82	0.27 (0.41)	67	0.30 (0.38)	0.901	0.881
Wheezing preceding 12 months	70	0.29 (0.41)	67	0.30 (0.38)	0.954	0.876
Rhinitis preceding 12 months	68	0.27 (0.41)	67	0.30 (0.38)	0.773	0.928
Eczema preceding 12 months	36	0.25 (0.37)	67	0.30 (0.38)	0.787	0.900

a Mann-Whitney *U*-test.

b Dunnett test on log-transformed data.

**Table 4:** Association between concentration of phthalates in dust and health outcomes in 184 children.

	No. [OR (95% CI)]				
	1st quartile (Ref)	2nd quartile	3rd quartile	4th quartile	*p*-Value^a^
DEHP crude analysis					
Range (mg/g)	0.02–0.41	0.42–0.99	1.00–2.29	2.30–29.45	
Case status	46 (1.0)	46 [1.3 (0.6–2.9)]	46 [1.4 (0.6–3.2)]	46 [2.7 (1.2–6.4)]	0.025
Wheezing preceding 12 months	41 (1.0)	42 [1.4 (0.6–3.4)]	44 [1.7 (0.7–4.0)]	43 [3.2 (1.3–7.9)]	0.010
Rhinitis preceding 12 months	44 (1.0)	44 [1.3 (0.6–3.0)]	45 [1.5 (0.6–3.5)]	35 [2.0 (0.8–4.9)]	0.132
Eczema preceding 12 months	36 (1.0)	33 [1.1 (0.4–3.1)]	29 [0.9 (0.3–2.5)]	28 [2.3 (0.8–6.3)]	0.190
DEHP adjusted analysis^b^					
Case status	46 (1.0)	46 [1.7 (0.7–4.3)]	43 [1.2 (0.5–3.0)]	46 [2.9 (1.1–7.5)]	0.058
Wheezing preceding 12 months	41 (1.0)	42 [2.0 (0.8–5.5)]	41 [1.5 (0.6–4.0)]	43 [3.7 (1.4–9.9)]	0.023
Rhinitis preceding 12 months	44 (1.0)	44 [1.6 (0.6–4.2)]	42 [1.2 (0.5–3.1)]	35 [2.1 (0.8–5.6)]	0.254
Eczema preceding 12 months	36 (1.0)	33 [1.5 (0.5–4.7)]	28 [0.7 (0.2–2.6)]	28 [2.6 (0.8–8.4)]	0.260

Ref, referent.

a *p*-Value for trend test. The DEHP variable was put into the logistic regression model as a continuous variable including the values 1, 2, 3, and 4.

b Analyses adjusted for age (< 3 years vs. 5–7 years), sex (male vs. female), smoking at home during pregnancy and first year of child’s life (at least one occupant smoking vs. no smoking at home), current smoking at home (at least one occupant smoking vs. no smoking at home), allergy or asthma in family (at least one symptomatic family member vs. no other person with symptoms).

**Table 5:** Measurements of the concentration of phthalates in dust in different countries.

			DEHP (mg/g dust)		BBzP (mg/g dust)		DnBP (mg/g dust)	
Study	Country	No.	50th^a^	95th^a^	50th^a^	95th^a^	50th^a^	95th^a^
Present study	Bulgaria	184	0.99	7.98	0.33	1.56	9.85	30.8
Bornehag et al. 2004	Sweden	346	0.77	4.07	0.13	0.60	0.15	0.57
Pohner et al. 1997	Germany	272	0.45	2.00	ND	ND	ND	ND
Oie et al. 1997	Norway	38	0.64^b^	—	0.11^b^	—	0.10^b^	—
Butte et al. 2001	Germany	286	0.74	2.60	0.05	0.32	0.05	0.24
Becker et al. 2002	Germany	199	0.42	1.19	0.01	0.21	0.04	0.16
Clausen et al. 2003	Denmark	23	0.86	2.59	ND	ND	ND	ND
Rudel et al. 2003	USA	120	0.34	0.85^c^	0.04	0.28^c^	0.02	0.04^c^
Kersten and Reich 2003	Germany	65	0.60	1.60	0.02	0.23	0.05	0.18
Fromme et al. 2004	Germany	30	0.70	1.54	0.03	0.22	0.06	0.13
Becker et al. 2004	Germany	252	0.51	1.84	ND	ND	ND	ND

ND, no data. Table adapted from Bornehag et al. (2005a).

a 50th and 95th percentiles.

b Mean concentration.

c 90th percentile.
